# Integration of animal health and public health surveillance sources to exhaustively inform the risk of zoonosis: An application to echinococcosis in Rio Negro, Argentina

**DOI:** 10.1371/journal.pntd.0008545

**Published:** 2020-08-25

**Authors:** Andrew Lawson, R Boaz, A. Corberán-Vallet, Marcos Arezo, Edmundo Larrieu, Marco A. Vigilato, Victor J. Del Rio Vilas

**Affiliations:** 1 Medical University of South Carolina, Charleston, South Carolina, United States of America; 2 University of Valencia, Valencia, Spain; 3 Ministerio de Salud, Viedma, Rio Negro, Argentina; 4 Universidad Nacional de Rio Negro, Chole Choel, Argentina; 5 Organización Panamericana de la Salud, San Salvador, El Salvador; 6 Centre for Universal Health, Chatham House, London, United Kingdom; Fundacao Oswaldo Cruz, BRAZIL

## Abstract

The analysis of zoonotic disease risk requires the consideration of both human and animal geo-referenced disease incidence data. Here we show an application of joint Bayesian analyses to the study of echinococcosis granulosus (EG) in the province of Rio Negro, Argentina. We focus on merging passive and active surveillance data sources of animal and human EG cases using joint Bayesian spatial and spatio-temporal models. While similar spatial clustering and temporal trending was apparent, there appears to be limited lagged dependence between animal and human outcomes. Beyond the data quality issues relating to missingness at different times, we were able to identify relations between dog and human data and the highest ‘at risk’ areas for echinococcosis within the province.

## Author summary

This work focuses on utilizing animal disease data to try and inform our understanding of the spread of diseases in humans. We implement predictive models to estimate the relationship between the distribution of disease in animal populations and the distribution of disease in human populations. Development of a better understanding of this relationship could inform animal and public health interventions aiming to mitigate against human disease before it spreads. Missing data and limited data resources made discovery of these relationships difficult, but we fit multiple model types to try and identify any connection between these two populations. We found specific areas with elevated risk of human disease and changes in disease risk over time. Finally, there was some indication of an association between previous years’ levels of animal disease and human disease when using animals as covariables.

## Introduction

Cystic echinococcosis (CE) is caused by *Echinococcus granulosus* (EG), a cestode of the family Taeniidae whose hosts are herbivore and carnivore animals (sheep and dogs being the most important involved in the transmission to man). It is a parasitic zoonosis endemic in South America, especially in Argentina, Chile, Peru, Uruguay and southern Brazil [1].

Among the factors that contribute to the transmission of EG, the type of sheep production in subsistence economies and the practice of feeding sheep offal to dogs appear most relevant. Other factors, such as temperature and humidity, impact the survival and dispersion of the parasite’s eggs to the environment [2].

**Fig 1:**
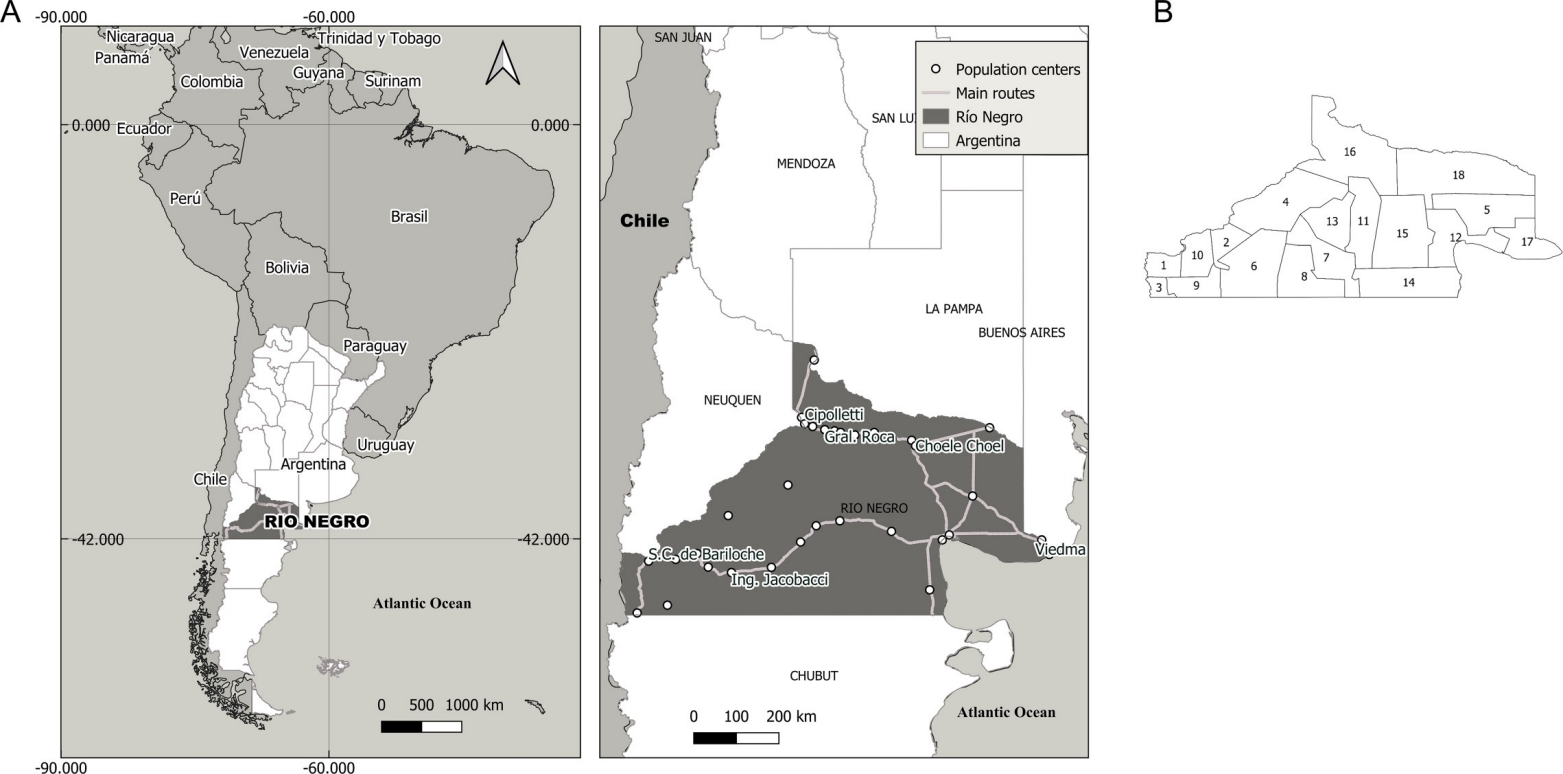
A) Location of the Rio Negro region study region within ARgentina and within the context of South America. B) Map of Rio Negro regions: Bariloche (1), Comallo (2), El bolson (3), El Cuy (4), Gral. Conesa (5), Jacobacci (6), Los Menucos (7), Maquinchao (8), Niorquinco (9), Pilcaniyeu (10), Ramos Mexia (11), San Antonio (12), Sierra Colorada (13), Sierra Grande (14), Valcheta (15), Valle Alto (16), Valle Inferior (17), Valle Medio (18).

In the province of Rio Negro, south of Argentina, CE is the most important zoonosis. In 1980, when the province’s control program against CE was launched, passive surveillance detected 146 human cases (incidence rate: 73 cases per 100,000). The location of the province of Rio Negro is highlighted in Fig 1A and its 18 subregions in Fig 1B. Rio Negro is one of the six provinces that make up the Argentine Patagonia. The CE endemic area is in the south-west of the province, in an area of 120,013 km2 with a population density of 0.88 inhabitants/ km2. The CE program control area has 13 hospitals and a network of 80 Primary Health Care Centers (PHCCs), normally manned by a sanitary agent or nurse in rural areas, and a general practitioner in urban PHCCs.

The geographic areas of greatest risk are located to the west and center of the province [3–5] including the towns of Bolsón and Bariloche in the Cordillera region and those of Comallo, Pilcaniyeu, Ñorquinco and Ingeniero Jacobacci and their rural areas in the Patagonian plateau region, where the ecological conditions favor the survival of Echinococcus granulosus eggs and the social, cultural and economic conditions, with subsistence sheep farming and the persistent practice of feeding sheep offal to the dogs, generate an epidemiological environment that favors the sustenance of the transmission cycle [6].

The control program, which mainly consisted of regular administration of praziquantel to dogs, contributed to the reduction of CE incidence to 29 cases per 100,000 in 1997, and to 7 cases per 100,000 by 2016. Surveillance is essential to evaluate the effectiveness of the control efforts. In addition to the clinical cases detected via passive surveillance, active surveillance in the form of ultrasound screening (US) of all 7 to 14 years old children attending school was implemented [7]. In 1984, the first US campaign showed a prevalence of 5.6% [7]. Subsequent screening in 1997 showed a prevalence of 1.1%, and of 0.2% in 2016 [7,8].

Surveys of sheep farms monitoring environmental exposure were also conducted, specifically targeting lambs and resident dogs to monitor recent infection. CoproELISA was used as a screening method in dogs with confirmation by Western Blot (WB) in fecal samples obtained from the environment [9,10]. ELISA was used as a screening test on sera from sheep with confirmation by WB [11].

Disease risk mapping is important for the understanding of the spatial epidemiology of infectious diseases. Integration of data and analyses, whether of population or health related variables, has been shown to improve risk classification accuracy and the efficacy of detection [12]. However, in most cases, even for multi-host diseases such as zoonoses, risk estimation has been conducted in an univariate fashion based on human case data alone. Specifically, for zoonotic diseases, knowledge of spatial and temporal patterns of the animal host could inform incidence in humans [13]. Linked models have been widely used in several diseases such as tularemia [13], and respiratory diseases [14]. Bayesian approaches have been used extensively to model disease counts at the small area level [15,16], and to facilitate the joint modeling of animal and human data [17].

For CE, Bayesian and spatio-temporal analysis models have been developed widely (e.g. in China [18, 19], Iran [20] and Kyrgyzstan [21]). However, none of these studies merged multiple surveillance sources and simultaneously combined animal and human case data. The aim of this study is to exhaustively analyze CE surveillance data for the identification of joint patterns of disease in humans and animals, and the achievement of more precise CE risk estimates in the province of Rio Negro, Argentina.

## Materials and methods

### Materials

#### Animal data

Sheep farms, selected following a simple random sampling scheme across the entire province, constituted our epidemiological unit (EU) of interest. Samples of canine faecal matter were collected from the soil of sheep farms in close proximity to the houses. Samples were processed by coproELISA and, if positive, confirmed with WB. In parallel, blood samples from lambs at the same EU were processed by ELISA with confirmation by WB. A positive sample classified the EU as having recent transmission [10, 22].

Two data sets were considered:

- Lamb and dog annual case data for the period 2003–06: It includes data on the total number of farms sampled and the total number of farms with recent transmission (at least one dog or one lamb positive).
- Dog case data for the year 2010: It includes data on the number of farms sampled and the number of farms with recent transmission (at least one dog positive).

#### Human data

Annual clinical cases detected by passive surveillance, were classified in children up to 14 years of age and adults, in the period 1997–2016. We chose this classification of clinical cases to allow comparisons of recent infections in children vs. old exposure in adults. Information was also collected from the cases diagnosed in US surveys of asymptomatic school children from 6 to 14 years old (active surveillance). All cases were georeferenced to their health program area [7].

Both animal and human data were aggregated at the health program area level (*m* = 18) in Rio Negro (Fig 1).

### Methods

#### Spatial analysis of animal data

In this first analysis, we consider the previously described animal data and fit a spatial model with the aim of estimating the risk of recent transmission in each health program area for the two periods 2003–06 and 2010.

#### Model formulation

Let *y*_*i*_ represent the number of farms with recent transmission out of those that have been sampled (*f*_*i*_) in the *i*th health program area. At the first level of the model hierarchy, we assume that the number of diseased farms *y*_*i*_ are described by a binomial distribution with *p*_*i*_ the probability of a farm having recent transmission in area *i*.

At the second level of the model hierarchy, the logit of the probability, *p*_*i*_, is decomposed in additive components representing spatial effects. In particular, we assume that the logit (*p_i_*) = *α*_0_+*u_i_+v_i_*, where *α*_0_ represents the overall risk of disease in the study region (here Rio Negro), and *u_i_,v_i_* are, respectively, spatially correlated and uncorrelated random effects. The prior distributions for these parameters are defined as zero mean Gaussian for the intercept and uncorrelated random effect, and a ICAR correlation model for ν_*i*_ [15]. Conventional prior distributions are assumed for the standard deviations of these effects. In particular, we assume a uniform distribution on a fixed range. This type of model is frequently used to provide an optimal description of the spatial variation of disease risk in spatial epidemiology studies [16].

Information was missing on both the number of cases and the number of farms sampled for certain years. We modelled this missingness by means of a Poisson distribution with parameter *λ_i_* which has a Gamma prior distribution defined to mirror the sampled population. The average number of farms sampled per area, 20.14, was used to set the mean of the Gamma prior distribution.

### Spatio-temporal analysis of animal data

In this section, we consider the annual data and fit a spatio-temporal model with the aim of estimating the risk of recent transmission in each area and its evolution over time.

#### Model formulation

As in the spatial analysis, we assume that the number of farms with recent transmission out of those that have been sampled in the *i*th small area at time *j* (*f*_*ij*_) follow a binomial distribution with *p*_*ij*_ the probability of a farm having recent transmission.

At the second level of the model hierarchy, the logit of the probability (*p*_*ij*_) is decomposed in additive components representing spatial and temporal effects:

$$logit(p_{ij})=a_{0}+u_{i}+v_{i}+\gamma_{j}+\delta_{ij}$$

where *α*_0_, *u*_*i*_ and *v*_*i*_ are defined as those above, *γ_j_* is the temporal random walk component representing a common trend in the study region, and *δ_ij_* is uncorrelated space-time interaction random effect. As done in the spatial models for missing data, we assume that *f_ij_* has a Poisson distribution with a parameter which has a suitably scaled gamma prior distribution. This prior sets the mean number of sampled farms as 15, which is similar to the number of farms sampled in the areas with complete information.

### Human case analysis

We jointly modelled active and passive surveillance data. In one scenario (see below), we split the active surveillance data into adult and child cases.

#### Model formulation

For Model 1 (and variants 1.1, 1.1.a, 1.1.b, and 1.2) we let *cases*(*all*)_*ij*_ represent the number of passive surveillance cases observed for both children and adults together, *e*(*all*)_*ij*_ represents the expected case count, *n*.*screened*_*ij*_ the total number of children screened (active surveillance), and *pos*.*screens*_*ij*_ the number of screened children with a positive test. The passive surveillance model is set up as a Poisson data model for all cases with mean *e*(*all*)_ij_*θ_ij_ where, *θ_ij_* represents the relative risk of surveillance cases in area *i* at time *j*.

The screening model on the other hand assumes that positive screening findings have a binomial distribution with probability *p_ij_*. Due to missingness in the screening data, we assume that we could use the screening observed in non-missing areas as a basis for imputation for missing areas. Missing *n*.*screened* data is imputed using a *Pois*(*λ_ij_*) distribution with *λ_ij_* having a gamma prior distribution that reflects the mean number of screened individuals for those health program areas with data (approximately 200). *θ*_*ij*_ and *p*_*ij*_ were modeled with log and logit link functions respectively with varying degrees of linkage between the models. The full list of models is given in S1 Table.

Model variants include *v* and *v1* terms which are both uncorrelated error terms independent from one another. Each have Gaussian prior distributions. Spatio-temporal error terms, ψ and ψ1, are also included in some models with Gaussian prior distributions. The common spatial error and temporal error terms will allow us to determine if the screening and passive surveillance case data are behaving similarly across regions and time. Each of the modification factors has a unique Gaussian prior distribution.

Model 1.0 uses effects unique for each of the outcomes. That is, none of the terms modeling *θ* are used in modeling *p*. Model 1.1 allows the spatial correlation random effect (*u*) to be common to both models with a factor (*φ*) modifying the proportion of positive cases’ spatial effect. This common spatial effect allow us to link the outcomes we see in the surveillance and screening data with a common spatial trend. Model 1.1a uses the same common spatial trend, and incorporates a common temporal term (*γ*) trend with a factor (*χ*) modifying the effect. Model 1.1b uses common spatial, temporal, and spatio-temporal error terms with a modification factor ρ introduced for the spatio-temporal term. Model 1.2 uses all common random effect terms but a different intercept, trying to completely connect the two sets of data, just scaling by a different intercept.

Model 2 scenario (with variants 2.0, 2.1, 2.1.a) splits the passive surveillance data into children and adults, where child cases have a Poisson distribution with mean *e*(*child*)_ij_*θ.c_ij_ *a*nd adult cases with mean *e*(*adult*)_ij_*θ.a_ij_. All terms are defined similarly to when data was aggregated across age groups. In this case, age-based population data was used to develop the expected rates for children and adults, respectively. Also, the models for child and adult surveillance are linked by a common spatio-temporal term, δ, that has a zero mean Gaussian prior distribution. S2 Table shows the models tested using the split passive surveillance data.

Models 2.0, 2.1, and 2.1a mirror the relationships shown in Models 1.0, 1.1, and 1.1a; however, instead of modeling *θ* as the relative risk of disease aggregated for both adults and children, we split children and adults into two separate models with two separate *θ*s. Model 2.0 uses separate random effect terms for each of the 3 models (*θ*.c, *θ*.a, and *p*). Model 2.1 uses a common spatially correlated random effect (*u*) across all 3 models with modification factors *φ* and *φ*1 scaling the effect from *θ*.c to *θ*.a and *p* respectively. Model 2.1a uses the common *u* term with its modification factors and also uses a common temporal random effect term (*γ*) with modification factors *χ*1 and *χ*2.

### Human and animal analysis

In this section, we used the animal data as a covariate in our analysis of the human data. We evaluated the animal data in two separate ways. For Model 1.3a, we looked at aggregated data for farms with dogs for 2003–2004, data for farms with lambs for 2004, 2005, and 2006, and data for farms with dogs for 2010; we used the aggregated lamb and dog data as a single covariate measure for each region. In Model 1.3b we used the dog (2003–2004) and lamb (2004–2006) surveillance data, where we had both the number of animals screened and the number of positive tests. The covariate was calculated by adding up all of the positive cases of dogs or lambs over 2003–2006 and dividing by the total number of dogs and lambs screened over the same time period. Not all periods and areas had available animal data and so model 1.3b has no 2010 data, so that covariate was dropped. Both models are the same as Model 1.1a but with the addition of the mentioned covariates. For Model 1.3a, *ld*_2006*i*_ is the percentage of farms detected after dog and lamb tested positive in each region from 2003–2006, and *ld*_2010*i*_ is percentage of farms detected after dogs tested positive in each region for 2010:

$$\begin{array}{l} log(\theta_{ij})=\alpha_{0}+u_{i}+v_{i}+\gamma_{j}+\psi_{ij}+\beta_{2006i}*ld_{2006i}+\beta_{2010i}*ld_{2010i}\\ logit(p_{ij})=\alpha_{1}+\varphi *u_{i}+v1_{i}+\chi *\gamma_{j}+\psi 1_{ij}+\omega 1*\beta_{2006i}*ld_{2006i}+\omega 2*\beta_{2010i}*ld_{2010i} \end{array}$$

In Model 1.3a we added *β*_2006*i*_ and *β*_2010*i*_ as coefficients for the effect of the covariates in the surveillance model. The regression coefficients were allowed to have spatially structured prior distributions so that different areas could respond differently to the effect of predictors. We assumed that the regression parameters (β_*i_) have spatially correlated ICAR prior distributions while the precisions have uniform prior distributions.

We also used the same *β*s for the screening model but with modification factors *ω*1 and *ω*2 to link the two models. In Model 1.3b we excluded *β*_2010*i*_ for both surveillance and screening models (not shown).

### Model fitting procedure

All models were fit using WinBUGS, with a burn-in of 100,000 iterations and a posterior sample of between 5,000 and 25,000 iterations. We checked convergence based on the Brooks Gelman Rubin (BGR) diagnostic statistic for deviance first, and then for each of the model parameters being estimated. Models were compared using the Deviance Information Criterion (DIC) and effective parameters (pD) to compare the models’ fits.

### Ethics statement

The data used in this study consists of counts of diseased animals and humans from small areas. The human data arises from passive and active surveillance. All cases are de-identified and only the aggregated counts of cases are used in the analysis. Confidentiality of the human cases is assured by this de-identification and the aggregation ensures that the addresses are anonymized and hence not reported. Ethical approval was received for use of the data from the Departo Zoonosis, Ministerio Salud, Provincia de Rio Negro.

## Results

### Spatial analysis of animal data

Tables 1 and 2 show the proportion of farms with present transmission, the posterior mean estimate of the probability of a farm having recent transmission in each program area, and its standard deviation for lamb and dog data for 2003–2006 and dog data for 2010, respectively. The corresponding maps are shown in S1 and S2 Figs, respectively. The DIC (and pD) of the model for dog and lamb data for the period 2003–06 is 81.442 (14.418), whereas that for the dog data for 2010 was 44.274 (5.118).

**Table 1:** Lamb and dog data 2003–2006. Proportion of farms with recent transmission, estimated probability of a farm having recent transmission, and standard deviation.

Prog.Area	N.f sampled	N.f recent transmission	Proportion	Estimated p	SD(p)
Bariloche	1	1	1.000	0.300	0.260
Comallo	33	3	0.091	0.099	0.045
El bolson	44	1	0.023	0.047	0.030
El cuy	66	14	0.212	0.197	0.048
Gral. Conesa	41	1	0.024	0.048	0.030
Jacobacci	64	6	0.094	0.099	0.035
Los Menucos	69	7	0.101	0.106	0.034
Maquinchao	37	8	0.216	0.201	0.062
Niorquinco	84	11	0.131	0.128	0.035
Pilcaniyeu	50	4	0.080	0.089	0.037
Ramos Mexia	22	0	0.000	0.053	0.040
San Antonio	49	3	0.061	0.074	0.034
Sierra Colorada	11	1	0.091	0.109	0.071
Sierra Grande	30	8	0.267	0.233	0.075
Valcheta	83	15	0.181	0.172	0.041
Valle Alto	13	0	0.000	0.061	0.048
Valle Inferior	10	3	0.300	0.223	0.115
Valle Medio	7	0	0.000	0.073	0.060

**Table 2:** Dogs only data 2010. Proportion of farms with recent transmission, estimated probability of a farm having recent transmission, and its standard deviation.

Prog.Area	N.f sampled	N.f trans	Proportion	Estimated p	SD(p)
Bariloche	5	0	0.000	0.130	0.061
Comallo	9	1	0.111	0.132	0.052
El bolson	40	8	0.200	0.168	0.052
El cuy	41	5	0.122	0.124	0.037
Gral. Conesa	NA	NA	NA	0.126	0.080
Jacobacci	44	8	0.182	0.153	0.044
Los Menucos	17	2	0.118	0.125	0.045
Maquinchao	9	2	0.222	0.156	0.071
Niorquinco	20	3	0.150	0.143	0.050
Pilcaniyeu	9	1	0.111	0.134	0.057
Ramos Mexia	4	0	0	0.113	0.049
San Antonio	2	0	0	0.118	0.062
Sierra Colorada	22	1	0.045	0.101	0.041
Sierra Grande	7	1	0.143	0.125	0.057
Valcheta	53	5	0.094	0.108	0.035
Valle Alto	NA	NA	NA	0.125	0.069
Valle Inferior	NA	NA	NA	0.131	0.092
Valle Medio	NA	NA	NA	0.122	0.069

For 2003–06 data, no strong spatial patterns emerge, but we see that El cuy, Sierra Grande, and Valcheta have over 15% of sampled farms with transmission (Bariloche has 100%; however, only 1 farm was sampled).

For 2010 dog data, again, no spatial patterns seem to emerge, but El bolson, Jacobacci, Maquinchao, and Niorquinco have over 15% of sampled farms with recent transmission. Because the affected areas have clearly changed over the years, a spatio-temporal analysis may be more revealing.

### Spatio-temporal analysis of animal data

**Fig 2:**
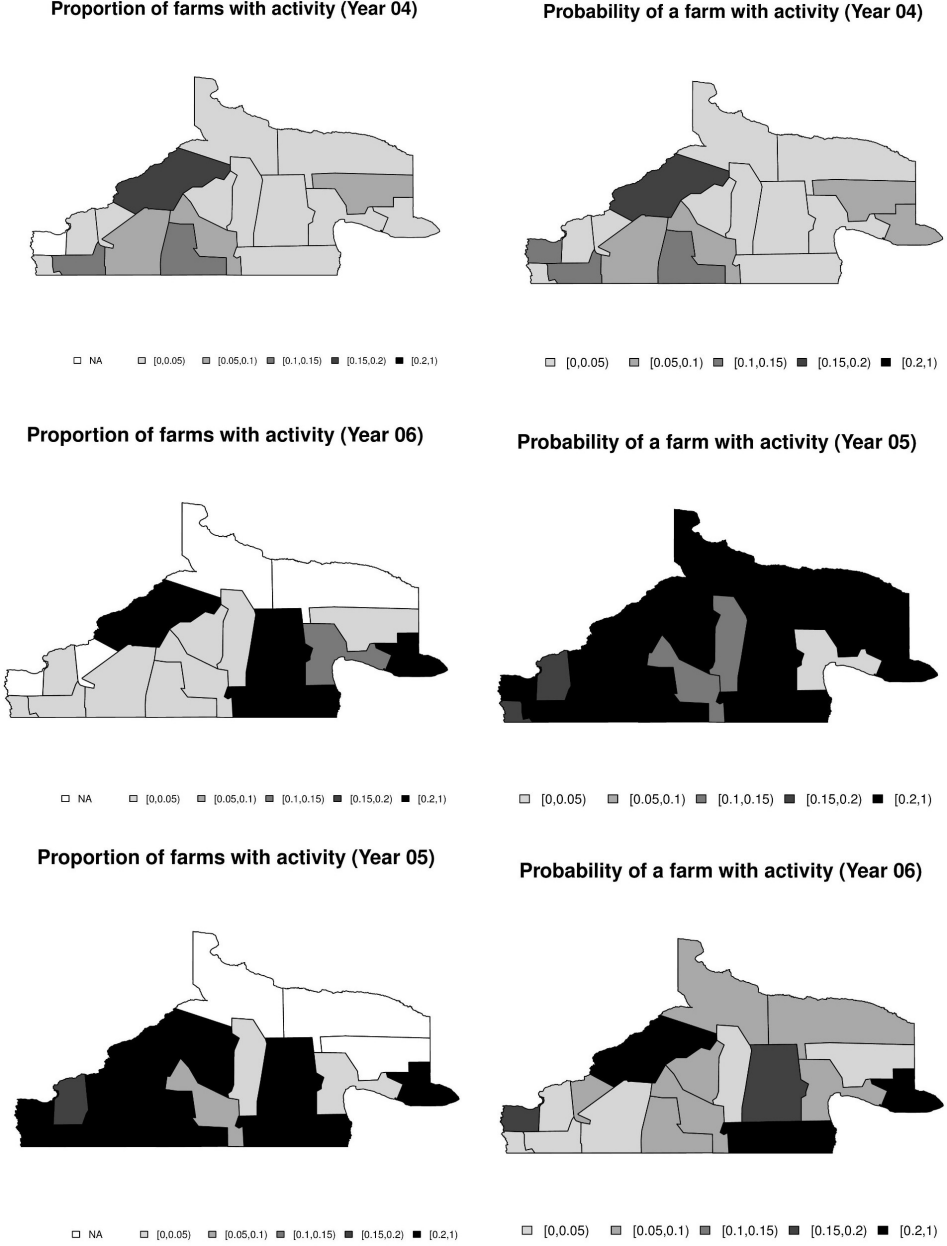
Annual data. Proportion of farms with recent transmission (left) and estimated probability of a farm having recent transmission (right).

S3 Table shows the annual proportion of farms with recent transmission and the posterior mean estimate of the probability of a farm having recent transmission in each program area. The corresponding maps are shown in Fig 2.

Here we see that in 2005 almost all regions showed >15% of sampled farms with transmission.

### Human case analysis

**Fig 3:**
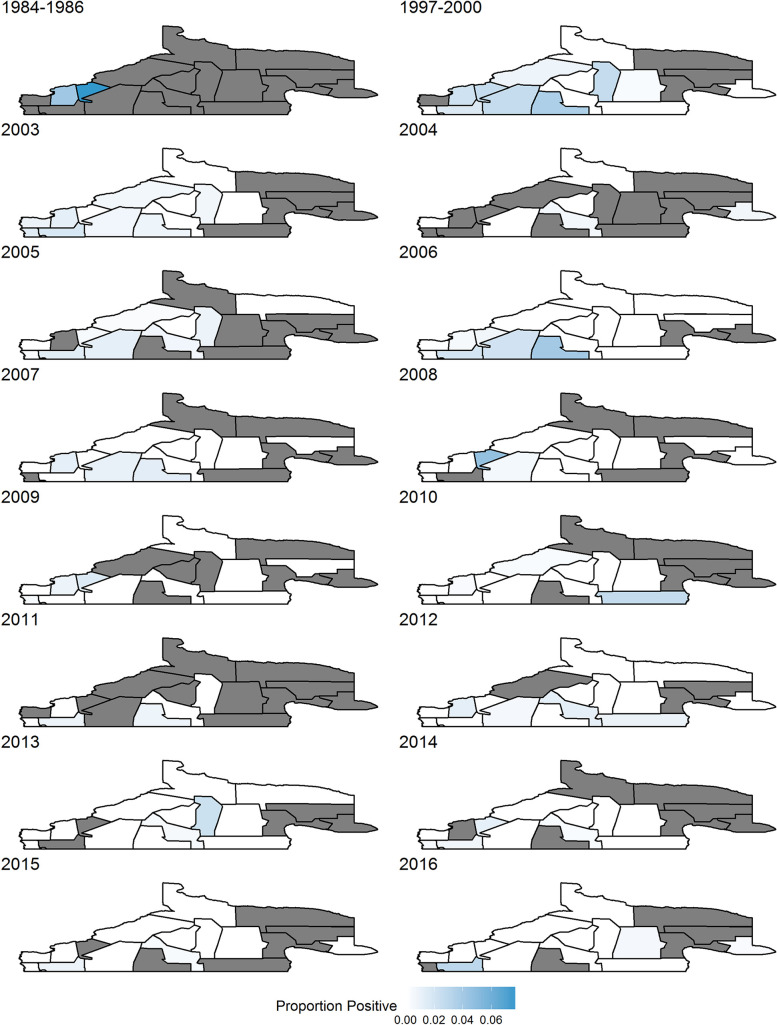
Proportion of screened people who were positive. Grey areas indicate no people were screened in those regions in that time frame.

Our next goal was to analyze human case data to identify when and where disease rates spike. Fig 3 shows the proportion of screened people who were positive for each region over all time periods: only aggregated data available for 1984–1986, and 1997–2000, whereas 2003–2016 has separate years. The southwest regions seem to be most affected, but rates are reducing over time as a whole.

In S3 Fig, rates of counts of disease divided by expected counts of disease for people is shown for years 2003–2016. Again, regions in the southwest show the highest rates, but some regions in the east were higher in early years, and regions in the central area show increased rates in later years. S4 Fig and S5 Fig represent rates when adult and child cases are taken separately. The child data is fairly sparse, and the human data mimics the overall data, with perhaps a greater skew to cases in the central areas.

#### Model comparison

Our models held different assumptions on the existence and strength of the association between human and animal data. We want to compare our model paradigms to investigate which is the best fit for our outcome data, providing some insight to the relationship between animals and humans. Because Model 1 aggregates all cases together and Model 2 splits the outcome into discrete categories of adult and child, the models cannot be compared using overall DIC. Within Model 1.0, 1.1, 1.1a, and 1.2 all DIC and pD values can be compared. Similarly, all DIC and pD values can be compared across models 2.0 and 2.1. Model 2.1a has a negative pD for outcome y2 (adult surveillance cases), which suggests a poor model fit and hence model mis-specification.

Model 1.1a is the best model, as its overall DIC is lowest and convergence was strong. It models passive surveillance data together (child and human), incorporates a common correlated spatial random effect and a common temporal random effect across both passive surveillance and screening data, modifying the effects by universal scaling factors *φ* and *χ*, respectively.

**Fig 4:**
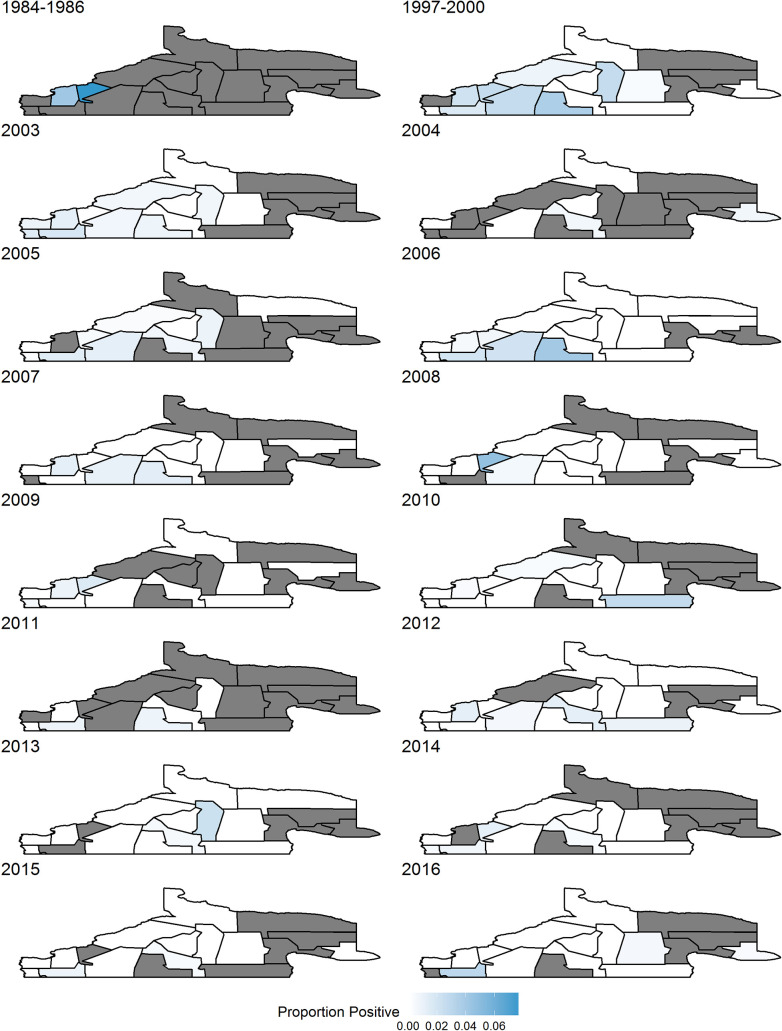
Model 1.1a - Spatially Correlated Error Term–*u*. Note that the values are zero centered and affect the relative risk on the log scale.

The modification factors *φ* and *χ* are estimated at close to 1 (0.668 and 1.286, respectively); however, their standard deviations are quite large (5.4 and 9.9, respectively). This suggests that the two surveillance streams (active and passive) are behaving similarly over space and time. In Fig 4, the correlated spatial error term (*u*) is shown in one map and suggest clustering of risk, and it is constant over all years. The southwest region again shows higher relative risk.

**Fig 5:**
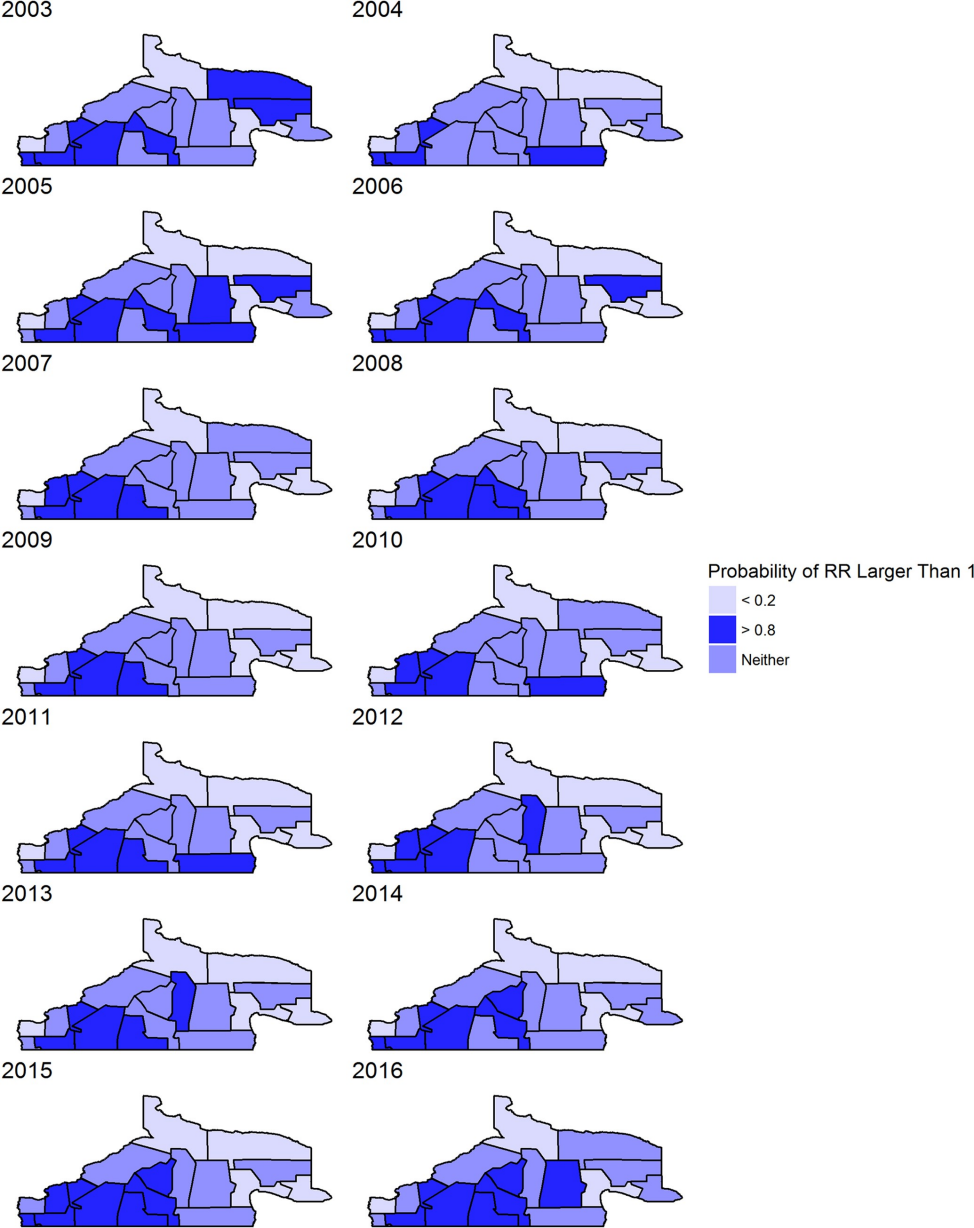
Posterior exceedences for relative risks from model 1.1a.

**Fig 6:**
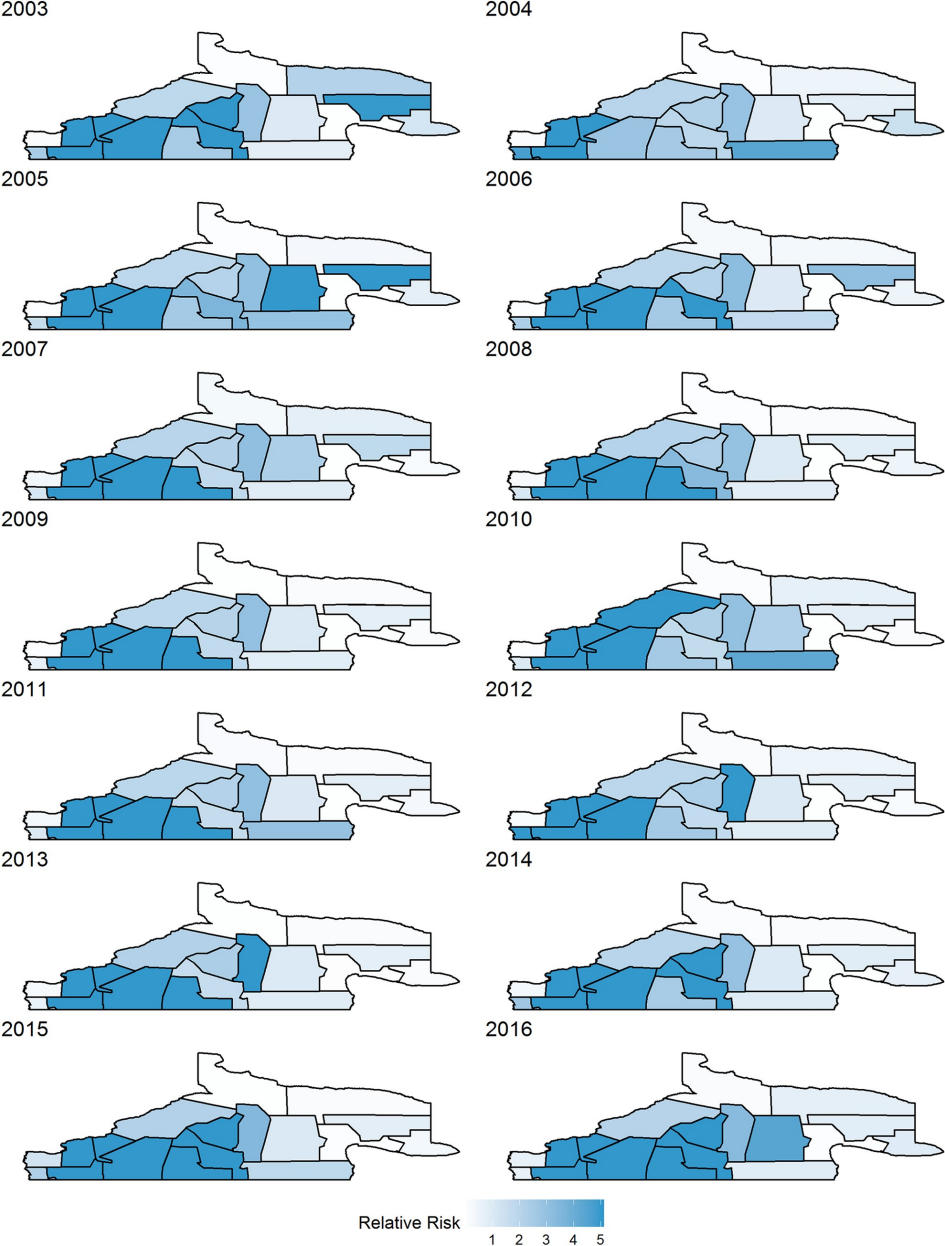
Model 1.1a –Posterior mean relative risks—Surveillance Data.

Fig 5 provides probabilities for program areas exceeding a RR of 1, that is P(RR>1), based on Model 1.1a. The figure splits regions into three categories: probability greater than 0.8, probability less than 0.2, and somewhere in between. These maps demonstrate where extreme risk estimates are located. These again show highest rates in the southwest, but the central regions showing higher values in more recent years. Note that any RR greater than 5 was set to 5 for legibility; many of the relative risks were much higher than 5 for these regions. Fig 6 displays the posterior mean estimates of risk and it is also evident that the south western areas are highest in disease risk.

### Human and animal analysis

**Fig 7:**
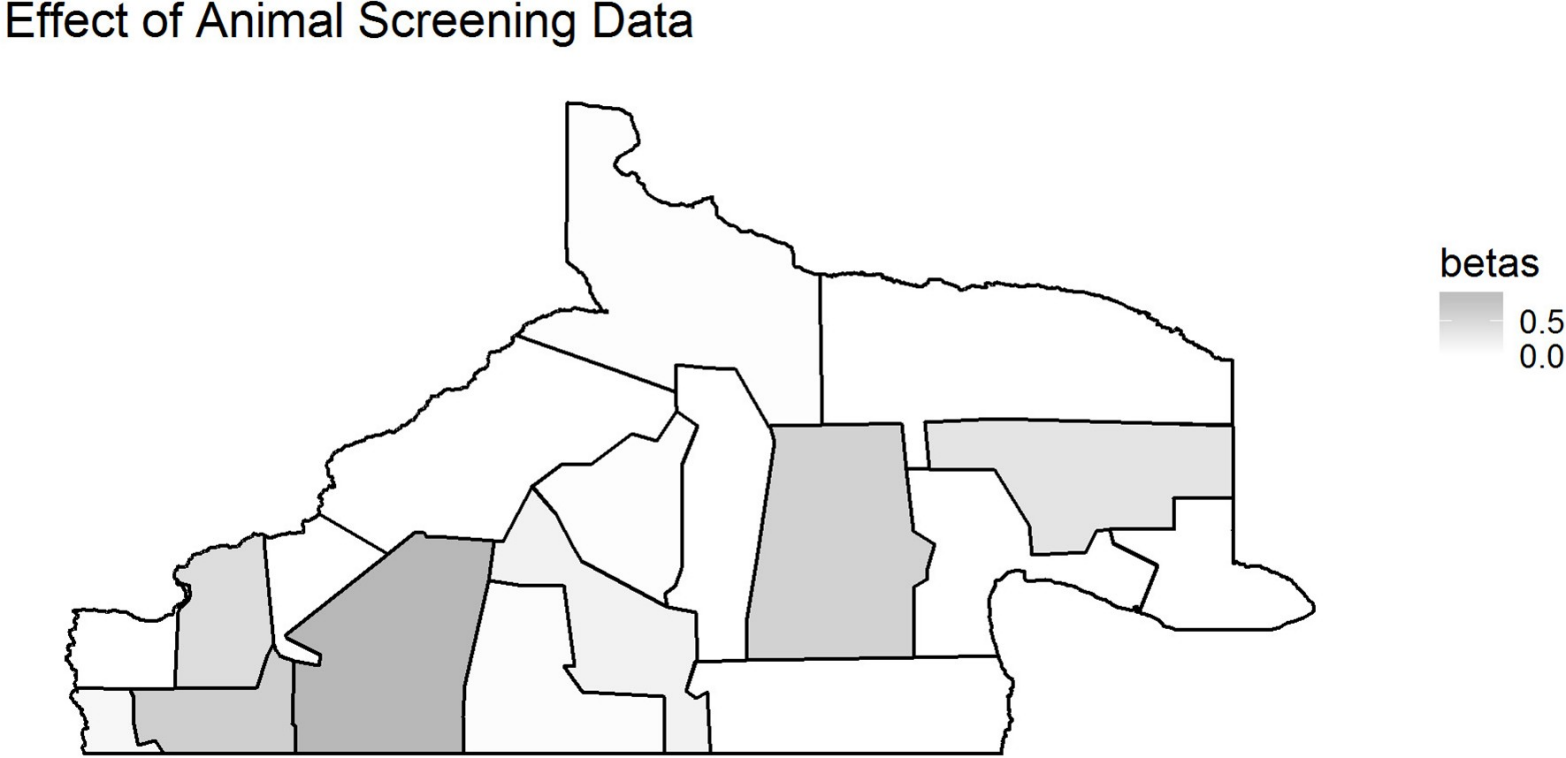
*β*_2006*i*_ values based on Model 1.3a.

**Fig 8:**
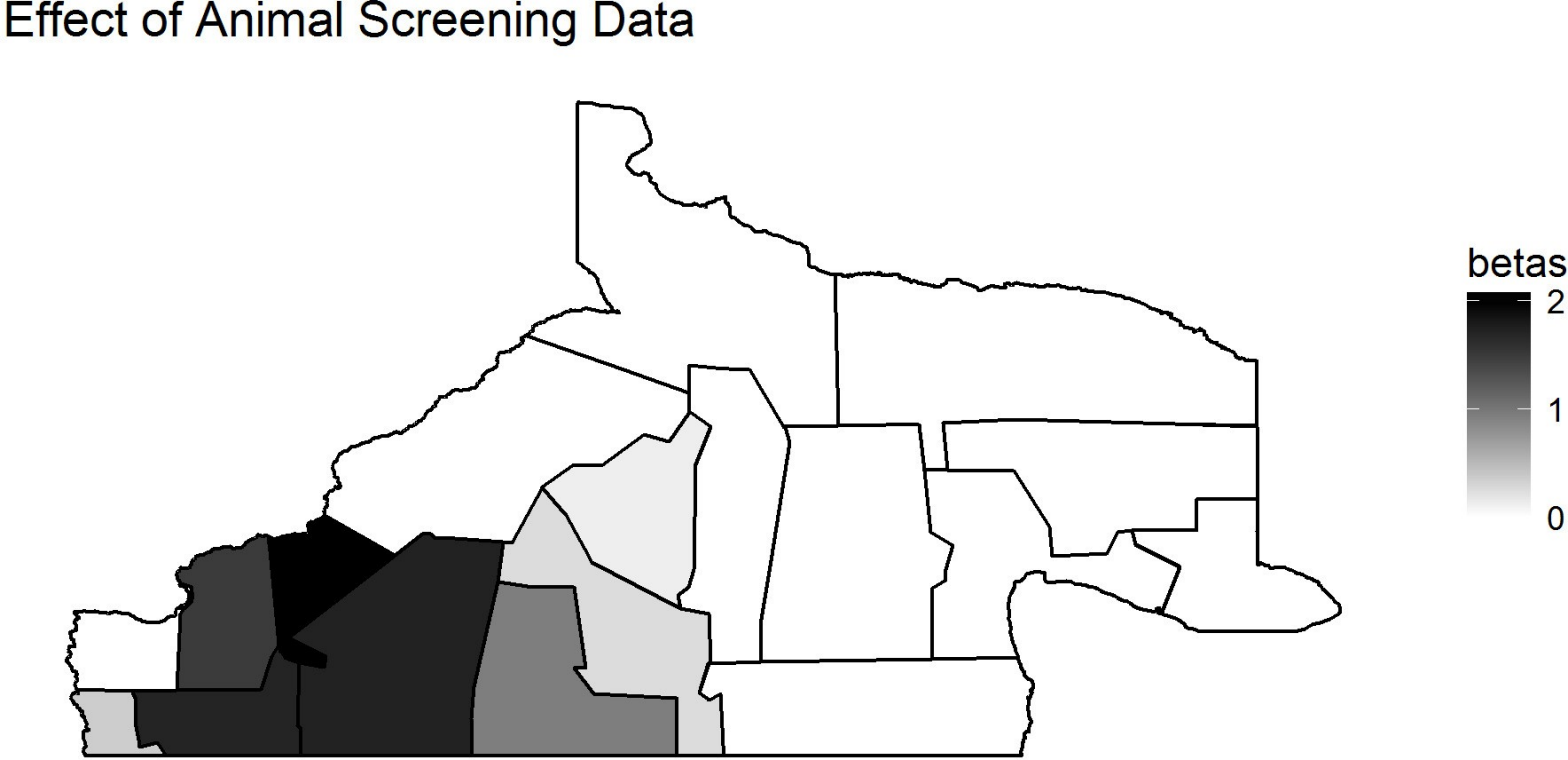
*β*_2010*i*_ values based on Model 1.3a.

Finally, we see results of using animal data as covariates in the human model. Fig 7 and Fig 8 show the program area-specific estimates of *β*_*2006i*_ and *β*_2010*i*_ based on Model 1.3a, showing the impact of the animal screening data, where positive values of *β* indicate that an increase in the percentage of positively screened animals results in an increased rate/count of human cases. Again, we see that the southwest has high values of *β*_2010*i*_, which may indicate that the rates of association between screening of animal data and human data are dependent upon where the disease is. However, we also ran Model 1.3c with a constant *β*_2006_ coefficient to see if there was an overall association, and we had a value of 0.47 with sd of 5.332, indicating it was not statistically significant for this model. It is of note that all β values are positive, indicating a positive relationship between the number of positively screened animals and the number of human cases.

## Discussion

The analysis of screening data and passive surveillance data showed that the most appropriate model was that with a common spatial and temporal trend for both types of data, indicating that both surveillance systems are capturing similar disease patterns across regions and timespans. This suggests there may not be a strong difference between the observed adult and child incidence of echinococcosis. The analysis of the human data while using the animal data as a covariate did not show any conclusive relationship for the passive and screening data models; however, the areas of most elevated human risk (southwest), also showed the strongest association with animal case data. S1 Fig shows that 2006 animal rates were not particularly clustered, and yet Fig 6 shows that there may still be a strong impact of animal disease rates on human disease rates in the southwest area. Animal disease does seem to be elevated in the southwest in 2010 (S2 Fig), but the elevated values of the coefficients for animal impact in the southwest, shown in Fig 7, continue to suggest that the rates of animal disease in this area are driving the elevated human impacts in the southwest shown in Figs 4 and 5. However, the lack of evidence for a constant β_2006_ suggests that the overall predictive impact of animal rates is not strong for human outcomes. The observed association between animal and human cases is likely to reflect ongoing long term transmission trends given the chronic nature of the clinical disease in humans

The southwest area’s elevated human rates and higher impact of animal disease may merit a finer scale case study of the transmission of disease between animals and humans, to account for the likely heterogeneity within health program areas.

In general, the geographical areas identified as being most at risk are those located in the west and center of the province, including Comallo, Pilcaniyeu, Ñorquinco and Ingeniero Jacobacci and their rural areas, in the Patagonian plateau region, coinciding with previous reports [10,22].

The CE control program in Rio Negro has maintained a regular activity since its inception in 1980, however with interruptions in the deployment of some surveillance streams throughout the years. As a result of this situation, the initial occurrence of CE in people, including children from 6 to 14 years old, has persistently decreased, but the transmission is maintained in some pockets. In this context, adjustments to the epidemiological surveillance system towards detection of such defined areas are warranted. Our analyses aim to provide the evidence to support such adjustments. Likewise, it is essential to identify the effectiveness of each of the surveillance systems applied, including their correlation and / or enhancement, in order to eventually reduce the operating costs of the system.

Surveillance in dogs, for its part, is the only tool to identify present transmission. With the advent of coproELISAs in the 1990s, laboratory testing of dog faeces was possible on a large scale and copro tests were used for surveillance in many programs, including Rio Negro [23].

### Conclusion

Due to the complexity of parasitic cycles such as that of EG (passage through the definitive host, environment, time of evolution in the human host and time of detection of infection) the association of animal-human surveillance sources becomes more difficult to characterise. Added to this is the difficulty in obtaining adequate data on animal surveillance in time and space, due to the geographical extension of control programs, the inherent associated logistical challenges, and limited operational capacity. Conducting studies at finer spatial scales and in smaller areas with high case rates where animal surveillance can be intensified may be an option for future research. Still, we were able to identify some relationship between animal and human data, specifically with the 2010 dog data. These results support a continued study of the predictive capabilities of animal disease data in understanding human risk.

### Limitations

While our study has exploited the available data across surveys and passive surveillance, and used state of the art methods to inform the transmission behavior of CE, there are a number of limitations. Primarily, the fragmented scope of the animal and human data has led to reduced ability to find equivalent data frames. This has also limited the ability to detect lagged effects. Differences in data quality also inevitably impact the inferential ability of any methods. In addition, the sparseness in terms of low or zero counts in some areas can lead to less certainty in estimation of rates. While CE outbreaks are affected by environmental factors such as humidity, temperature and rainfall, we have endeavored to assess the animal–human linkage by accounting for confounding partially generated by these factors. It may be that inclusion of these factors could also help to elucidate the animal-human linkage. This we would examine in future work.

## Supporting information

S1 Table Human spatio-temporal analysis models using aggregated surveillance data. (DOCX)

S2 Table Models tested using surveillance data split by child and adult. (DOCX)

S3 Table Spatio-temporal analysis of Lamb and dog 2004–06. Annual proportion of farms with recent transmission and estimated probability of a farm having recent transmission.
(DOCX)

S1 Fig Lamb and dog 2003–06. Proportion of farms with recent transmission and estimated probability of a farm having recent transmission.
(TIFF)

S2 Fig Dogs 2010. Proportion of farms with recent transmission and estimated probability of a farm having recent transmission.
(TIFF)

S3 Fig Rates of Counts/Expected Counts for Humans (Surveillance Data). Counts are sum of human and child counts of disease, and missing data was reported as 0 cases for that region/year. Expected cases were calculated based on total population of each region during that year.
(TIFF)

S4 Fig Rates of Counts/Expected Counts for Adults (15+). (TIFF)

S5 Fig Rates of Counts/Expected Counts for Children (0–14). Note that maps with ALL PURPLE is the result of no reported cases for children in those years (2003, 2005, and 2015).
(TIFF)

S6 Fig Model 1.1a - Probability of Exceedance—Surveillance Data. (TIFF)
