# Supplementary material for: Integration of animal health and public health surveillance sources to exhaustively inform the risk of zoonosis: An application to echinococcosis in Rio Negro, Argentina
Source: PLoS Negl Trop Dis. 2020 Aug 25;14(8):e0008545. doi: 10.1371/journal.pntd.0008545 (PMC7473527; doi:10.1371/journal.pntd.0008545)
Supplement: S1 Table — (DOCX) [file pntd.0008545.s001.docx]

Supplemental Table 1. Human spatio-temporal analysis models using aggregated surveillance data.

| Model # | Model(s) | Model Notes |
| --- | --- | --- |
| 1.0 | 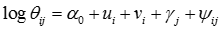 | Surveillance counts of adults and children aggregated – No common terms. |
|  | 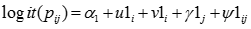 |  |
| 1.1 | 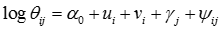 | Surveillance counts of adults and children aggregated; separate Surveillance and Screening Models EXCEPT common correlated spatial term (*u*) with a modification factor φ. |
|  | 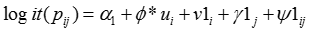 |  |
| 1.1a | 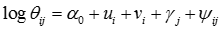 | Surveillance counts of adults and children aggregated; Separate Surveillance and Screening Models EXCEPT common correlated spatial term (*u*) with a modification factor φ AND common temporal term (γ) with a modification factor χ. |
|  | 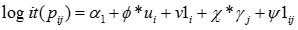 |  |
| 1.1b | 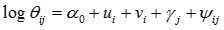 | Surveillance counts of adults and children aggregated; Separate Surveillance and Screening Models EXCEPT common correlated spatial term (*u*) with a modification factor φ AND common temporal term (γ) with a modification factor χ AND a common space-time interaction term (ψ) with a modification factor ρ. |
|  | 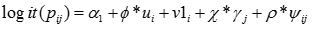 |  |
| 1.2 | 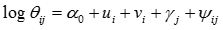 | Surveillance counts of adults and children aggregated; ALL common terms in the models EXCEPT intercept (α) |
|  | 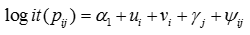 |  |
