# Supplementary material for: Integration of animal health and public health surveillance sources to exhaustively inform the risk of zoonosis: An application to echinococcosis in Rio Negro, Argentina
Source: PLoS Negl Trop Dis. 2020 Aug 25;14(8):e0008545. doi: 10.1371/journal.pntd.0008545 (PMC7473527; doi:10.1371/journal.pntd.0008545)
Supplement: S2 Table — (DOCX) [file pntd.0008545.s002.docx]

Supplemental Table 2. Models tested using surveillance data split by child and adult.

| Model # | Model(s) | Model Notes |
| --- | --- | --- |
| 2.0 | 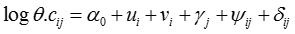 | Separate models for child and adult Surveillance Data. Child and adult surveillance models are linked by common spatio-temporal error term: δ. |
|  | 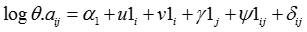 |  |
|  | 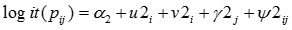 |  |
| 2.1 | 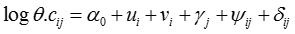 | Separate models for child and adult Surveillance Data; Separate models EXCEPT common correlated spatial term (*u*) across all 3 models with modification factors φ and φ1 |
|  | 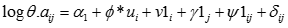 |  |
|  | 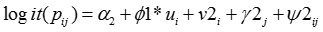 |  |
| 2.1a | 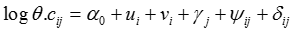 | Separate models for child and adult Surveillance Data; Separate models EXCEPT common correlated spatial term (*u*) across all 3 models with modification factors φ and φ 1AND common temporal term (γ) with modification factors χ and χ 1 |
|  | 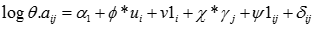 |  |
|  | 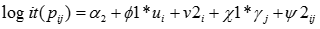 |  |
