# Supplementary material for: Integration of animal health and public health surveillance sources to exhaustively inform the risk of zoonosis: An application to echinococcosis in Rio Negro, Argentina
Source: PLoS Negl Trop Dis. 2020 Aug 25;14(8):e0008545. doi: 10.1371/journal.pntd.0008545 (PMC7473527; doi:10.1371/journal.pntd.0008545)
Supplement: S3 Table — Annual proportion of farms with recent transmission and estimated probability of a farm having recent transmission. (DOCX) [file pntd.0008545.s003.docx]

Supplemental Table 3: Spatio-temporal analysis of Lamb and dog 2004-06. Annual proportion of farms with recent transmission and estimated probability of a farm having recent transmission

| Prog.Area | Prop 04 | Prop 05 | Prop 06 | Est p 04 | Est p 05 | Est p 06 |
| --- | --- | --- | --- | --- | --- | --- |
| Gral. Conesa | 0.062 | NA | 0.000 | 0.050 | 0.299 | 0.021 |
| Bariloche | NA | 1.000 | NA | 0.134 | 0.563 | 0.168 |
| Pilcaniyeu | 0.043 | 0.167 | 0.000 | 0.038 | 0.190 | 0.022 |
| El bolson | 0.000 | 0.200 | 0.000 | 0.013 | 0.193 | 0.027 |
| Niorquinco | 0.148 | 0.333 | 0.000 | 0.123 | 0.327 | 0.020 |
| Jacobacci | 0.087 | 0.500 | 0.000 | 0.080 | 0.412 | 0.043 |
| Comallo | 0.000 | 0.300 | NA | 0.019 | 0.287 | 0.098 |
| El cuy | 0.172 | 0.333 | 0.250 | 0.156 | 0.364 | 0.235 |
| Maquinchao | 0.138 | 0.667 | 0.000 | 0.125 | 0.554 | 0.071 |
| Los Menucos | 0.072 | 0.062 | 0.000 | 0.067 | 0.102 | 0.079 |
| Sierra Grande | 0.000 | 0.833 | 0.250 | 0.039 | 0.714 | 0.216 |
| Sierra Colorada | 0.000 | 1.000 | 0.000 | 0.035 | 0.532 | 0.096 |
| Valcheta | 0.023 | 0.471 | 0.222 | 0.032 | 0.457 | 0.193 |
| Ramos Mexia | 0.000 | 0.000 | 0.000 | 0.018 | 0.103 | 0.040 |
| San Antonio | 0.000 | 0.000 | 0.100 | 0.029 | 0.040 | 0.080 |
| Valle Inferior | 0.000 | 1.000 | 1.000 | 0.063 | 0.638 | 0.524 |
| Valle Alto | 0.000 | NA | NA | 0.024 | 0.294 | 0.098 |
| Valle Medio | 0.000 | NA | NA | 0.027 | 0.294 | 0.099 |
