# Supplementary figures and images for: Integration of animal health and public health surveillance sources to exhaustively inform the risk of zoonosis: An application to echinococcosis in Rio Negro, Argentina
Source: PLoS Negl Trop Dis. 2020 Aug 25;14(8):e0008545. doi: 10.1371/journal.pntd.0008545 (PMC7473527; doi:10.1371/journal.pntd.0008545)

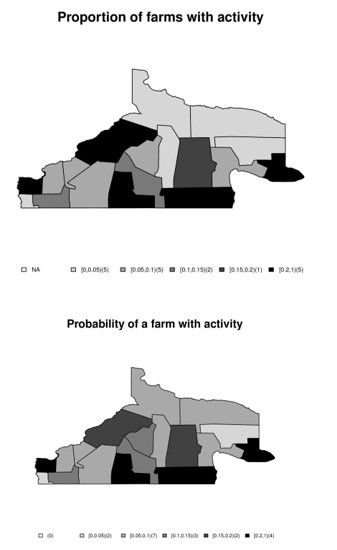

Supplement: S1 Fig — Proportion of farms with recent transmission and estimated probability of a farm having recent transmission. (TIFF) [file pntd.0008545.s004.tiff]

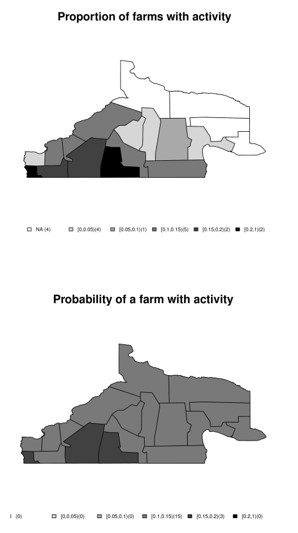

Supplement: S2 Fig — Proportion of farms with recent transmission and estimated probability of a farm having recent transmission. (TIFF) [file pntd.0008545.s005.tiff]

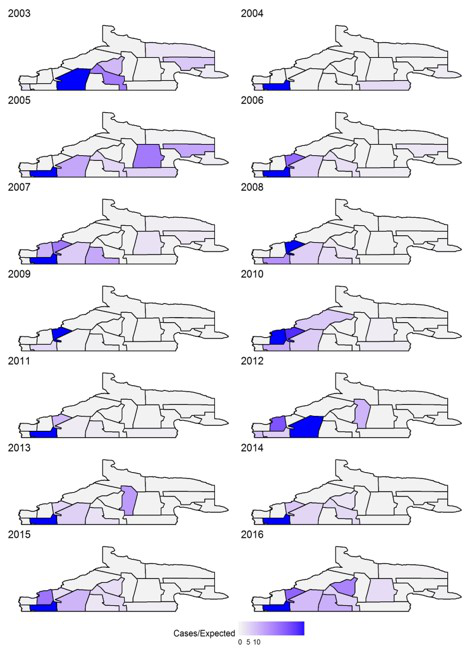

Supplement: S3 Fig — Counts are sum of human and child counts of disease, and missing data was reported as 0 cases for that region/year. Expected cases were calculated based on total population of each region during that year. (TIFF) [file pntd.0008545.s006.tiff]

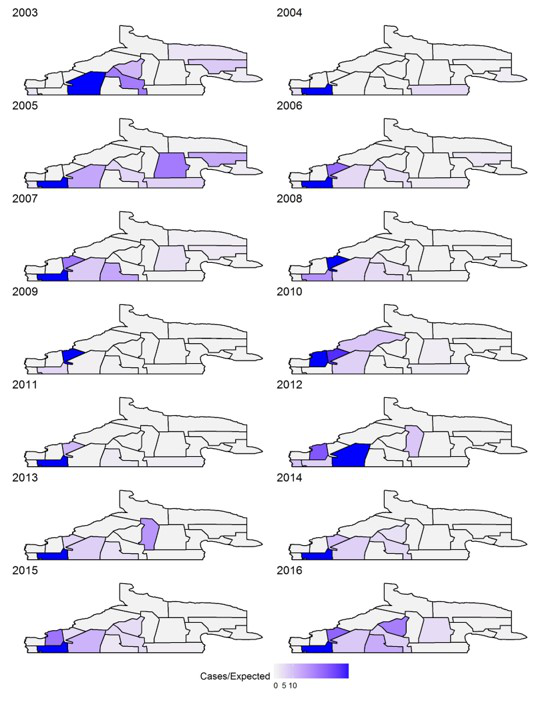

Supplement: S4 Fig — (TIFF) [file pntd.0008545.s007.tiff]

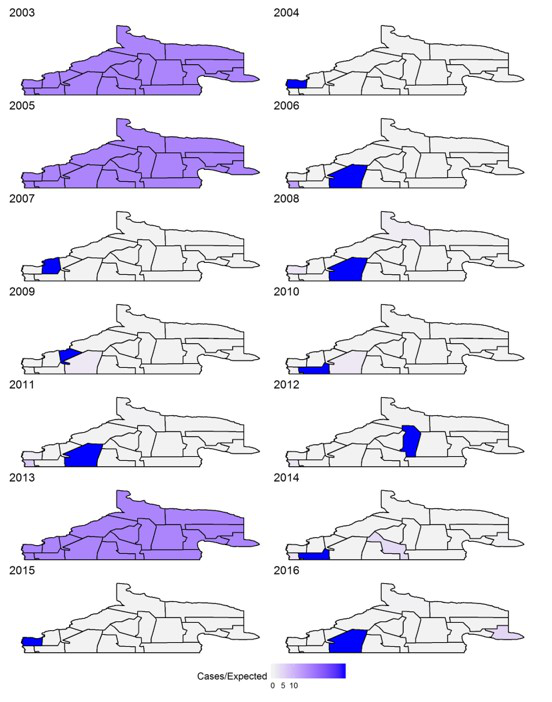

Supplement: S5 Fig — Note that maps with ALL PURPLE is the result of no reported cases for children in those years (2003, 2005, and 2015). (TIFF) [file pntd.0008545.s008.tiff]

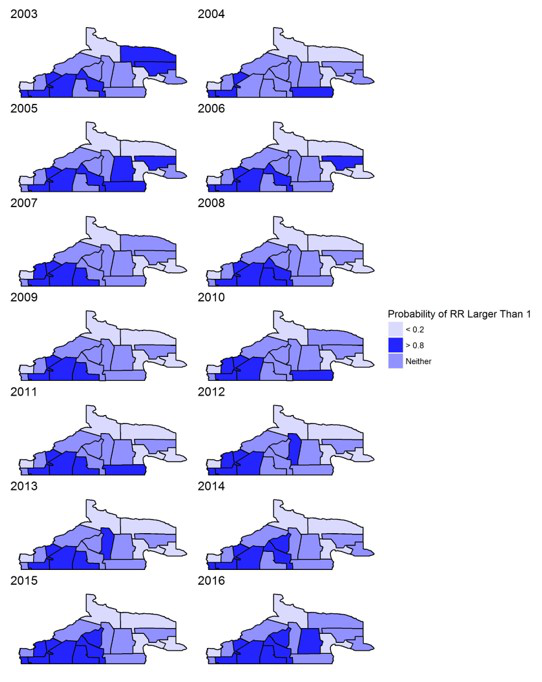

Supplement: S6 Fig — (TIFF) [file pntd.0008545.s009.tiff]
